# A universal AutoScore framework to develop interpretable scoring systems for predicting common types of clinical outcomes

**DOI:** 10.1016/j.xpro.2023.102302

**Published:** 2023-05-12

**Authors:** Feng Xie, Yilin Ning, Mingxuan Liu, Siqi Li, Seyed Ehsan Saffari, Han Yuan, Victor Volovici, Daniel Shu Wei Ting, Benjamin Alan Goldstein, Marcus Eng Hock Ong, Roger Vaughan, Bibhas Chakraborty, Nan Liu

**Affiliations:** 1 Centre for Quantitative Medicine, Duke-NUS Medical School, Singapore 169857, Singapore; 2 Programme in Health Services and Systems Research, Duke-NUS Medical School, Singapore 169857, Singapore; 3 Department of Neurosurgery, Erasmus MC University Medical Center, 3015 GD Rotterdam, the Netherlands; 4 Department of Public Health, Erasmus MC, 3015 GD Rotterdam, the Netherlands; 5 Singapore Eye Research Institute, Singapore National Eye Centre, Singapore 168751, Singapore; 6 SingHealth AI Office, Singapore Health Services, Singapore 168582, Singapore; 7 Department of Biostatistics and Bioinformatics, Duke University, Durham, NC 27710, USA; 8 Health Services Research Centre, Singapore Health Services, Singapore 169856, Singapore; 9 Department of Emergency Medicine, Singapore General Hospital, Singapore 169608, Singapore; 10 Department of Statistics and Data Science, National University of Singapore, Singapore 117546, Singapore; 11 Institute of Data Science, National University of Singapore, Singapore 117602, Singapore

**Keywords:** Health Sciences, Computer sciences

## Abstract

The AutoScore framework can automatically generate data-driven clinical scores in various clinical applications. Here, we present a protocol for developing clinical scoring systems for binary, survival, and ordinal outcomes using the open-source AutoScore package. We describe steps for package installation, detailed data processing and checking, and variable ranking. We then explain how to iterate through steps for variable selection, score generation, fine-tuning, and evaluation to generate understandable and explainable scoring systems using data-driven evidence and clinical knowledge.

For complete details on the use and execution of this protocol, please refer to Xie et al. (2020),^1^ Xie et al. (2022)^2^, Saffari et al. (2022)^3^ and the online tutorial https://nliulab.github.io/AutoScore/.

## Highlights

• A machine learning framework for automated development of clinical risk scores
• A common workflow to handle binary, survival, and ordinal outcomes
• Detailed demonstration of R package usage using publicly shared clinical data
• Rich statistics and visualizations to facilitate model evaluation and application

## Abstract (editor highlights)

Publisher’s note: Undertaking any experimental protocol requires adherence to local institutional guidelines for laboratory safety and ethics.

## Graphical abstract

**Figure:**
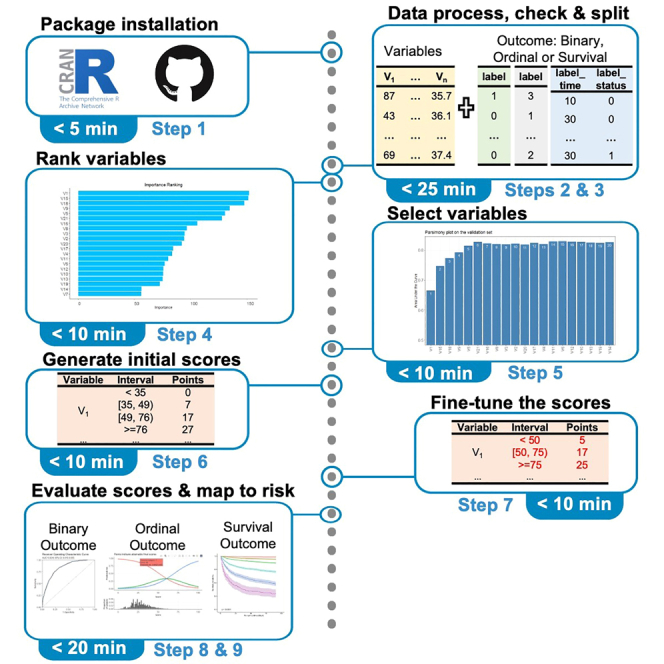

## Before you begin

Scoring systems are widely used in clinical settings for the convenient assessment of individual risk and may provide an easy-to-use tool to underpin clinical decision-making.^4^^,^^5^^,^^6^^,^^7^ For example, the well-known Framingham hypertension risk score uses seven routinely collected variables to identify high-risk patients for early intervention and efficient management, including lifestyle change programs and blood pressure-lowering treatment.^8^^,^^9^ The LACE index^10^ uses basic information on current inpatient stay, previous visits to emergency departments and comorbidity to identify patients with an elevated risk of adverse outcomes post-discharge for additional care. With the increased availability of data and analytical tools, there have been ongoing efforts to update existing scores^11^ and to devise new clinical risk scores for a wide range of clinical applications.^4^^,^^12^

Scoring systems are inherently easily interpretable, as they represent linear classification models that only require users to add, subtract and multiply a few numbers to make a prediction.^13^ The facile interpretability may support clinical decision-making, where doctors can easily understand in which risk category an individual patient falls.^14^^,^^15^^,^^16^^,^^17^ Compared with complex post-hoc explanations in machine learning, clinicians prefer intrinsically interpretable and transparent models, especially those used at the bedside.^18^^,^^19^^,^^20^^,^^21^

AutoScore^1^ was developed as an interpretable machine learning-based automatic clinical score generator. The framework consists of six modules: (1) variable ranking with machine learning, (2) variable transformation, (3) score derivation, (4) model selection, (5) domain knowledge-based score fine-tuning, and (6) performance evaluation. Using AutoScore, users can easily generate data-driven clinical scores while concomitantly incorporating clinical expertise and practical considerations.^22^^,^^23^^,^^24^^,^^25^^,^^26^ Besides binary outcomes,^1^ AutoScore has been methodologically extended to survival outcomes,^2^ unbalanced binary data^27^ and ordinal outcomes.^3^ The modularized structure allows AutoScore to be integrated with more advanced interpretable machine learning methods (e.g., the Shapley variable importance cloud^28^) for improved robustness, interpretability and transparency in the risk score development.^29^

This protocol demonstrates the unified AutoScore framework for developing interpretable scoring systems for three common types of clinical outcomes: binary, survival and ordinal, which has been implemented as an easy-to-use R package.^30^ This protocol is accompanied by an open-source codebase and simulated datasets demonstrating the whole score generation process. The protocol provides step-by-step instructions for users with diverse backgrounds (and possibly limited experience in programming) to conveniently develop scoring systems in different applications.

### Software prerequisites and data requirement

Before launching AutoScore, pre-installed R (>=3.5.0)^31^ and other R packages described in the key resources table are required. Detailed prerequisites and sample data format can be found in our online guidebook (https://nliulab.github.io/AutoScore/).

This protocol can be applied to tabular static data with binary, ordinal or survival outcomes; each demonstrated using a simulated clinical dataset with 20,000 samples. The example outcomes were inpatient mortality, a 3-category compound indicator of long inpatient stay and inpatient mortality, and 90-day survival in the intensive care unit, respectively, with simulated information on patient demographics, vital signs, and laboratory tests. AutoScore expects the input data to be complete without missing entries. Under certain circumstances, missing values in predictors (but not the outcome) may be automatically processed by AutoScore as an additional category. Still, instructions must be followed to check the data for missingness, as detailed in our online guidebook. This protocol focuses on the AutoScore application for complete data.

### Prepare a clinical question

Users should prepare a valid clinical question by consulting with clinicians and health professionals.^32^ Users should ensure that the target outcome is well-defined (either computationally using existing information or through manual labeling of the training dataset) and that data is available on clinically relevant predictors for the outcome. It is also important to identify who the likely end users will be and, thus, the most appropriate potential channels for the model output.^32^^,^^33^^,^^34^ Early engagement with an end-user group (e.g., practicing clinicians) can help refine the research question and identify real-world clinical pathways. This ensures that the model outputs can be ultimately seamlessly integrated into existing clinical workflows.

## Key resources table

**Table:** 

REAGENT or RESOURCE	SOURCE	IDENTIFIER
**Software and algorithms**		

R (>=3.5.0)	The R Foundation	RRID: SCR_001905 https://www.r-project.org
Rstudio	RStudio Team	RRID: SCR_000432 https://www.rstudio.com/
AutoScore	This paper	https://github.com/nliulab/AutoScore https://nliulab.github.io/AutoScore/ https://CRAN.R-project.org/package=AutoScore
randomForest	The R Foundation	https://CRAN.R-project.org/package=randomForest
randomForestSRC	The R Foundation	https://CRAN.R-project.org/package=randomForestSRC
survival	The R Foundation	https://CRAN.R-project.org/package=survival
ordinal	The R Foundation	https://CRAN.R-project.org/package=ordinal
pROC	The R Foundation	https://CRAN.R-project.org/package=pROC
coxed	The R Foundation	https://CRAN.R-project.org/package=coxed
Hmisc	The R Foundation	https://CRAN.R-project.org/package=Hmisc
survAUC	The R Foundation	https://CRAN.R-project.org/package=survAUC
survminer	The R Foundation	https://CRAN.R-project.org/package=survminer
tableone	The R Foundation	https://CRAN.R-project.org/package=tableone
car	The R Foundation	https://CRAN.R-project.org/package=car
dplyr	The R Foundation	https://CRAN.R-project.org/package=dplyr
tidyr	The R Foundation	https://CRAN.R-project.org/package=tidyr
magrittr	The R Foundation	https://CRAN.R-project.org/package=magrittr
rlang	The R Foundation	https://CRAN.R-project.org/package=rlang
knitr	The R Foundation	https://CRAN.R-project.org/package=knitr
ggplot2	The R Foundation	https://CRAN.R-project.org/package=ggplot2
plotly	The R Foundation	https://CRAN.R-project.org/package=plotly

## Step-by-step method details

As detailed in this section, the AutoScore framework is implemented in several general steps. We use Roman Numbers (i.e., (i), (ii), etc.) to denote general AutoScore steps, which often consist of several protocol steps (indicated by digits 1, 2, etc.). Table 1 provides an overview of AutoScore steps and corresponding functions in the R package, and in the following subsections, we will describe the installation instruction and usage.

**Table 1:** Definitions and arguments for key functions in AutoScore package

Functions			
Name(s)	Arguments	Usage	Descriptions
**Pipeline functions**			

AutoScore_rank() AutoScore_rank_Survival() AutoScore_rank_Ordinal()	train_set	A processed data.frame that contains data to be analyzed, for training.	AutoScore STEP(i): Rank variables with machine learning (AutoScore Module 1)
	ntree	Number of trees in the random forest (Default: 100).	
	method <binary only>	Method for ranking. Options: 1. ‘rf‘ – random forest-based ranking (default), 2. ‘auc‘ – AUC-based ranking (required validation set). For "auc", univariate models will be built based on the train set, and the variable ranking is constructed via the AUC performance of corresponding univariate models on the validation set (‘validation_set‘).	
	validation_set <binary only>	A processed data.frame that contains data to be analyzed, only for AUC-based ranking.	
AutoScore_parsimony() AutoScore_parsimony_Survival() AutoScore_parsimony_Ordinal()	train_set	A processed data.frame that contains data to be analyzed, for training.	AutoScore STEP(ii): Select the best model with parsimony plot (AutoScore Modules 2 + 3+4)
	validation_set	A processed data.frame that contains data for validation purpose.	
	rank	The ranking result generated from AutoScore STEP(i).	
	max_score	Maximum total score (Default: 100).	
	n_min	Minimum number of selected variables (Default: 1).	
	n_max	Maximum number of selected variables; default 20.	
	cross_validation	If set to TRUE, cross-validation would be used for generating parsimony plot, which is suitable for small-size data. Default to FALSE.	
	fold	The number of folds used in cross validation (Default: 10). Available if cross_validation = TRUE.	
	categorize	Methods for categorize continuous variables. Options include "quantile" or "kmeans" (Default: "quantile").	
	quantiles	Predefined quantiles to convert continuous variables to categorical ones. (Default: c(0, 0.05, 0.2, 0.8, 0.95, 1)) Available if categorize = "quantile".	
	max_cluster	The max number of cluster (Default: 5). Available if categorize = "kmeans".	
	do_trace	If set to TRUE, all results based on each fold of cross-validation would be printed out and plotted (Default: FALSE). Available if cross_validation = TRUE.	
	auc_lim_min	Min y_axis limit in the parsimony plot (Default: 0.5).	
	auc_lim_max	Max y_axis limit in the parsimony plot (Default: "adaptive").	
	link <ordinal only>	The link function used to model ordinal outcomes. Default is "logit" for proportional odds model. Other options are "cloglog" (proportional hazards model) and "probit".	
AutoScore_weighting() AutoScore_weighting_Survival() AutoScore_weighting_Ordinal()	train_set	A processed data.frame that contains data to be analyzed, for training.	AutoScore STEP(iii): Generate the initial score with the final list of variables (Re-run AutoScore Modules 2 + 3)
	validation_set	A processed data.frame that contains data for validation purpose.	
	final_variables	A vector containing the list of selected variables, selected from step(ii)AutoScore_parsimony. Run vignette("Guide_book", package = "AutoScore") to see the guidebook or vignette.	
	max_score	Maximum total score (Default: 100).	
	categorize	Methods for categorize continuous variables. Options include "quantile" or "kmeans" (Default: "quantile").	
	max_cluster	The max number of cluster (Default: 5). Available if categorize = "kmeans".	
	quantiles	Predefined quantiles to convert continuous variables to categorical ones. (Default: c(0, 0.05, 0.2, 0.8, 0.95, 1)) Available if categorize = "quantile".	
	metrics_ci <binary only>	Whether to calculate confidence interval for the metrics of sensitivity, specificity, etc.	
	time_point <survival only>	The time points to be evaluated using time-dependent AUC(t).	
	n_boot <ordinal only>	Number of bootstrap cycles to compute 95% CI for performance metrics.	
	link <ordinal only>	The link function used to model ordinal outcomes. Default is "logit" for proportional odds model. Other options are "cloglog" (proportional hazards model) and "probit".	
AutoScore_fine_tuning() AutoScore_fine_tuning_Survival() AutoScore_fine_tuning_Ordinal()	train_set	A processed data.frame that contains data to be analyzed, for training.	AutoScore STEP(iv): Fine-tune the score by revising cut_vec with domain knowledge (AutoScore Module 5)
	validation_set	A processed data.frame that contains data for validation purpose.	
	final_variables	A vector containing the list of selected variables, selected from step(ii) AutoScore_parsimony.	
	cut_vec	Generated from STEP(iii).	
	max_score	Maximum total score (Default: 100).	
	metrics_ci <binary only>	Whether to calculate confidence interval for the metrics of sensitivity, specificity, etc.	
	time_point <survival only>	The time points to be evaluated using time-dependent AUC(t).	
	n_boot <ordinal only>	Number of bootstrap cycles to compute 95% CI for performance metrics.	
	report_cindex <ordinal only>	Whether to report generalized c-index for model evaluation (Default:FALSE for faster evaluation).	
AutoScore_testing() AutoScore_testing_Survival() AutoScore_testing_Ordinal()	test_set	A processed data.frame that contains data for testing purpose. This data.frame should have same format as train_set (same variable names and outcomes).	AutoScore STEP(v): Evaluate the final score with ROC analysis (AutoScore Module 6)
	final_variables	A vector containing the list of selected variables, selected from Step(ii) AutoScore_parsimony. Run vignette("Guide_book", package = "AutoScore") to see the guidebook or vignette.	
	cut_vec	Generated from STEP(iii).	
	scoring_table	The final scoring table after fine-tuning, generated from STEP(iv).	
	threshold	Score threshold for the ROC analysis to generate sensitivity, specificity, etc. If set to "best", the optimal threshold will be calculated (Default: "best").	
	with_label	Set to TRUE if there are labels in the test_set and performance will be evaluated accordingly (Default:TRUE).	
	metrics_ci <binary only>	Whether to calculate confidence interval for the metrics of sensitivity, specificity, etc.	
	time_point <survival only>	The time points to be evaluated using time-dependent AUC(t).	
	n_boot <ordinal only>	Number of bootstrap cycles to compute 95% CI for performance metrics.	
**Optional functions**			

compute_descriptive_table()	df	A data.frame after checking and fulfilling the requirement of AutoScore.	Compute descriptive table for the dataset.
compute_uni_variable_table() compute_uni_variable_table_survival() compute_uni_variable_table_ordinal()	df	A data.frame after checking and fulfilling the requirement of AutoScore.	Create the table of univariable analysis
	link <ordinal only>	The link function used to model ordinal outcomes. Default is "logit" for proportional odds model. Other options are "cloglog" (proportional hazards model) and "probit".	
	n_digits <ordinal only>	Number of digits to print for estimated effect (Default:3).	
compute_multi_variable_table() compute_multi_variable_table_survival() compute_multi_variable_table_ordinal()	df	A data.frame, which should have passed check_data().	Generate the table of multivariable analysis for your dataset.
	link <ordinal only>	The link function used to model ordinal outcomes. Default is "logit" for proportional odds model. Other options are "cloglog" (proportional hazards model) and "probit".	
	n_digits <ordinal only>	Number of digits to print for exponentiated coefficients (OR if logit link is used) (Default:3).	
conversion_table()	pred_score	A vector with outcomes and final scores generated from AutoScore_testing() .	Plot conversion table for binary outcomes based on final performance evaluation
	by	Specify correct method for categorizing the threshold: by "risk" or "score". Default to "risk".	
	values	A vector of threshold for analyze sensitivity, specificity and other metrics. Default to "c(0.01,0.05,0.1,0.2,0.5)".	
conversion_table_survival()	pred_score	A data frame with outcomes and final scores generated from AutoScore_testing_Survival().	Plot conversion table for survival outcomes
	score_cut	Score cut-offs to be used for generating conversion table; default c(40, 50, 60).	
	time_point	The time points to be evaluated using time-dependent AUC(t).	
conversion_table_ordinal()	pred_score	A data.frame with outcomes and final scores generated from AutoScore_testing_Ordinal().	Plot conversion table for ordinal outcomes
	link	The link function used to model ordinal outcomes. Default is "logit" for proportional odds model. Other options are "cloglog" (proportional hazards model) and "probit".	
	max_score	Maximum attainable value of final scores.	
	score_breaks	A vector of score breaks to group scores. The average predicted risk will be reported for each score interval in the lookup table. Users are advised to first visualize the predicted risk for all attainable scores to determine scores (see plot_predicted_risk).	
plot_predicted_risk()	pred_score	Output from AutoScore_testing() or AutoScore_testing_Ordinal().	Plot predicted risk for binary and ordinal outcomes
	link <ordinal only>	The link function used in ordinal regression, which must be the same as the value used to build the risk score. Default is "logit" for proportional odds model.	
	max_score	Maximum total score (Default: 100).	
	final_variables	A vector containing the list of selected variables, selected from Step(ii).	
	scoring_table	The final scoring table after fine-tuning, generated from STEP(iv) by AutoScore_fine_tuning() or AutoScore_fine_tuning_Ordinal().	
	point_size	Size of points in the plot. Default is 0.5.	
plot_survival_km()	pred_score	Generated from STEP(v) AutoScore_testing_Survival().	Plot Kaplan-Meier (KM) curve for survival outcomes
	score_cut	Score cut-offs to be used for the analysis, default c(40, 50, 60).	
	risk.table	Allowed values include: TRUE or FALSE specifying whether to show or not the risk table. Default is TRUE.	
	title	Title displayed in the KM curve.	
	legend.title	Legend title displayed in the KM curve.	
	xlim	Limit for x, default c(0, 90).	
	break.x.by	Threshold for analyze sensitivity.	

### Install the package and the prerequisites

**Timing: < 5 min**

This step describes how to install the AutoScore package, which automatically installs all dependencies in the key resources table.

1. Install the stable version of AutoScore from CRAN:

> install.packages("AutoScore")

or the latest (development) version from GitHub:

> install.packages("devtools") # If not already installed

> library(devtools)

> install_github(repo = "nliulab/AutoScore", build_vignettes = TRUE)

**CRITICAL:** The commands above automatically install all dependencies of AutoScore (see the key resources table). Troubleshooting 1 suggests a solution to possible installation errors.

### Data processing and checking

**Timing: < 15 min**

This step checks and processes data to meet all requirements. AutoScore has specific requirements on the outcome, predictors and missing values.

2. Load data.
  a. Read data from CSV or Excel files.
  b. For this demo, use the integrated sample datasets in the package.

> library(AutoScore)

> data("sample_data") # Load data with binary outcome

> data("sample_data_survival") # Load data with survival outcome

> data("sample_data_ordinal") # Load data with ordinal outcome

**CRITICAL:** These sample datasets are simulated to demonstrate the workflow. Any results and scoring systems described in this protocol are created solely for the demonstration of AutoScore usage and may not be clinically meaningful. Variable names are intentionally masked to avoid misinterpretation and misuse of data and models.

***Note:*** These sample datasets used <500MB memory when loaded in R and generally consumed <1GB memory in the processing steps to be described below. Troubleshooting 2 discusses how to monitor memory usage and handle possible issues in subsequent steps when working with larger clinical datasets.

3. Check outcomes.
  a. For binary and ordinal outcomes, change the name of the outcome to “label” and make sure that no other variables use this name. The code below changes the name of the binary outcome in “sample_data” from “Mortality_inpatient” to “label”:
    > names(sample_data)[names(sample_data) == "Mortality_inpatient"] <- "label"
  b. For survival outcomes, change outcome names for the time variable and censoring status to “label_time” and “label_status”, respectively, and make sure that no other variables use these names.
    ***Note:*** Binary outcomes and censoring status of survival outcomes should be coded as “factor” data type with two categories, and ordinal outcomes should be “factor” with three or more categories. The following functions check data requirements for different types of outcomes:
    > check_data(sample_data) # For binary outcomes
    > check_data_ordinal(sample_data_ordinal) # For ordinal outcomes
    > check_data_survival(sample_data_survival) # For survival outcomes

4. Check variables.
  The functions “check_data()”, “check_data_survival()” and “check_data_ordinal()” demonstrated above also check whether predictors in the data fulfill the following requirements:
  a. No special characters are available in variable names, e.g., “[“, “]”, “(“, “)”, “,”. (Suggest using “_” to replace them if needed).
  b. The name of variables should be unique and not entirely included in other variable names.
  c. Independent variables should be numeric (class: “numeric“ or ”integer”) or categorical (class: “factor” or “logical”).
    **CRITICAL:** All data problems reported by “check_data()”, “check_data_survival()” or “check_data_ordinal()” must be fully resolved before proceeding to the modeling phase. Troubleshooting 3 and 4 elaborate on common data problems and suggested solutions.

5. Check missing values.
  The functions “check_data()”, “check_data_survival()” and “check_data_ordinal()” will report missing rates for any variable with missing entries (coded as “NA” in R):
  a. AutoScore expects the input dataset to be complete with no missing values. Users can proceed with modeling if the data is complete and fulfill other requirements described in steps 3 and 4.
  b. If there are missing values in the dataset and users believe the missingness is informative and prevalent enough to be preserved as “NA” rather than excluded or imputed, users can proceed with modeling because AutoScore can automatically handle missing values by treating them as a new category named “Unknown”.
  c. Otherwise, users should handle missing values using appropriate methods (e.g., imputation or complete data analysis) before proceeding with modeling.
    **CRITICAL:** If feasible, users are highly recommended to carefully handle missing values in the input dataset during data pre-processing and provide a complete dataset without missing values to AutoScore.
    ***Note:*** When imputing missing values or treating them as a new category, high missing rates (e.g., >80%) may reduce model stability and should be handled with caution. For simplicity, in this protocol, we only demonstrate sample data with complete information, and interested users can refer to Demo 3 in Chapters 4 to 6 in our online guidebook (https://nliulab.github.io/AutoScore/) for more details on data with missing values.

6. Optional operations.
  a. Check variable distribution.
  b. Handle outliers.

***Note:*** The raw electronic health records data may contain outliers caused by system errors or clerical mistakes. Users are recommended to handle them appropriately before using AutoScore to ensure optimal modeling performance.

### Splitting data

**Timing: < 10 min**

This step aims to randomly split the dataset into three separate datasets (training, validation, and test datasets) for model training, validation and testing.

7. Split the dataset into training, validation, and test datasets.

> set.seed(4)

> out_split <- split_data(data = sample_data, ratio = c(0.7, 0.1, 0.2))

> train_set <- out_split$train_set

> validation_set <- out_split$validation_set

> test_set <- out_split$test_set

***Note:*** The split-sample approach demonstrated above is suitable when there is a sufficient sample size, e.g., 20,000 observations in “sample_data”. AutoScore provides a cross-validation option for small sample sizes (see https://nliulab.github.io/AutoScore/). Users can skip this step if the three datasets have been prepared and have passed the check operations in the previous subsection.

### AutoScore step (i): Generate a variable ranking list

**Timing: < 10 min (depending on your data and computer)**

This is the first step of the AutoScore workflow, which uses machine learning algorithms to identify the top-ranking predictors for subsequent score generation.

***Note:*** From this step onwards, we describe R commands and outputs for the example with a binary outcome and provide additional information regarding survival and ordinal outcomes in Note.

8. To rank all current candidate variables, run the following command:

**Figure 1:**
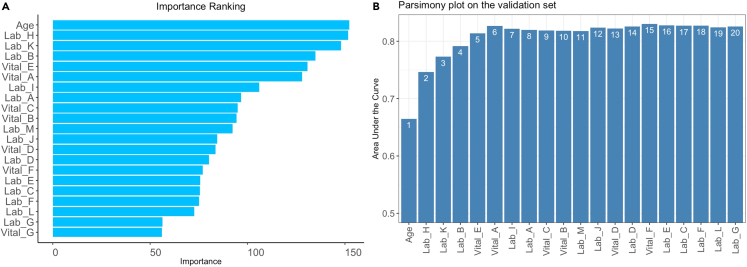
Main AutoScore output for variable ranking and selection (A) Variable importance from step 8 and (B) parsimony plot from step 9.

**Figure 2:**
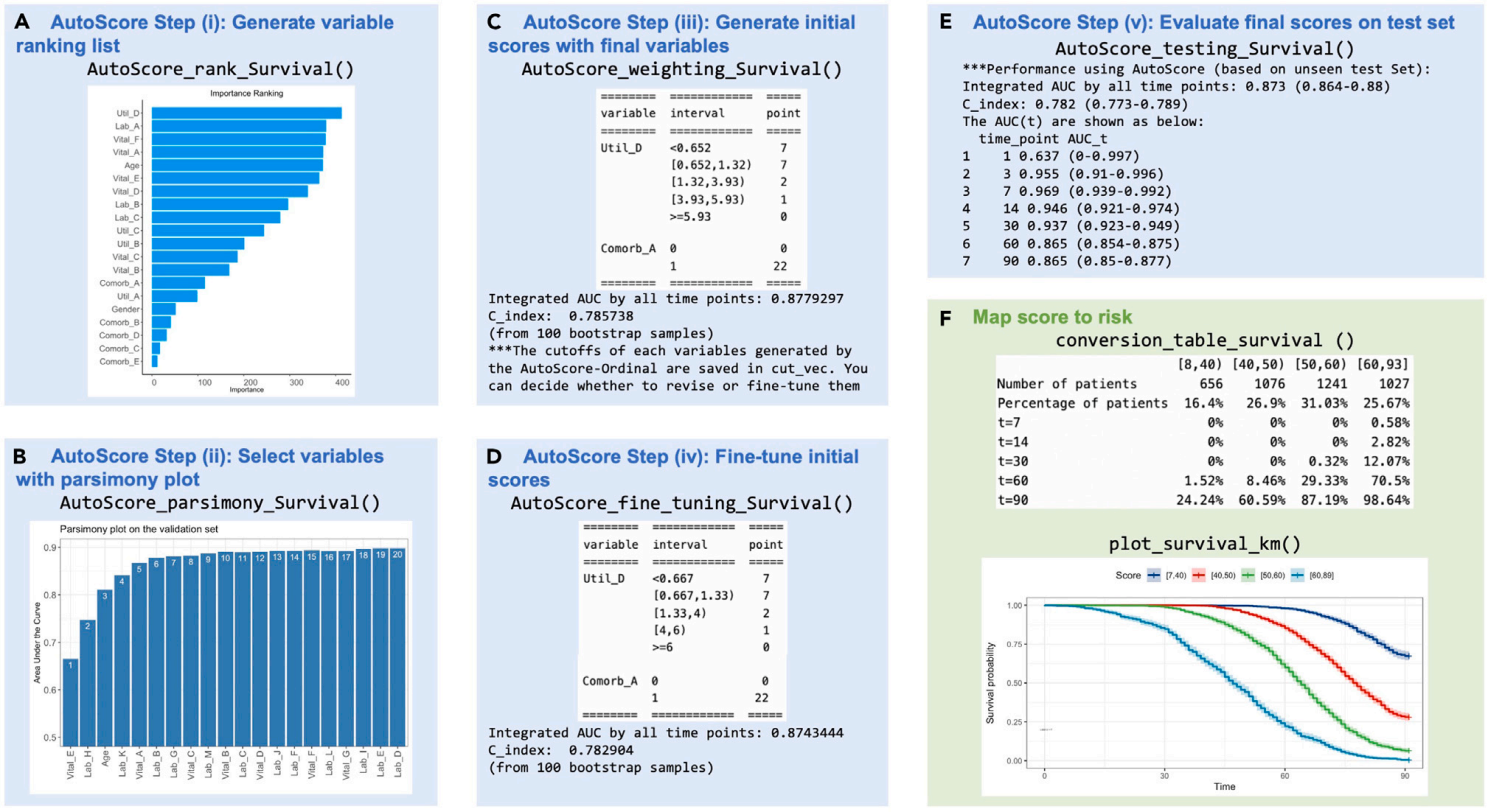
Main AutoScore functions for survival outcomes and corresponding output for score development and evaluation These sample datasets are simulated to demonstrate the workflow and any results and scoring systems described here are created solely for the demonstration. (A) Variable importance from step 8, (B) parsimony plot step 9, (C) initial scoring table and performance measures from step 11, (D) fine-tuned scoring table and performance measures from step 13, (E) performance measure of the final scoring model from step 14, and (F) conversion table and visualization of predicted probabilities from steps 15 and 16.

**Figure 3:**
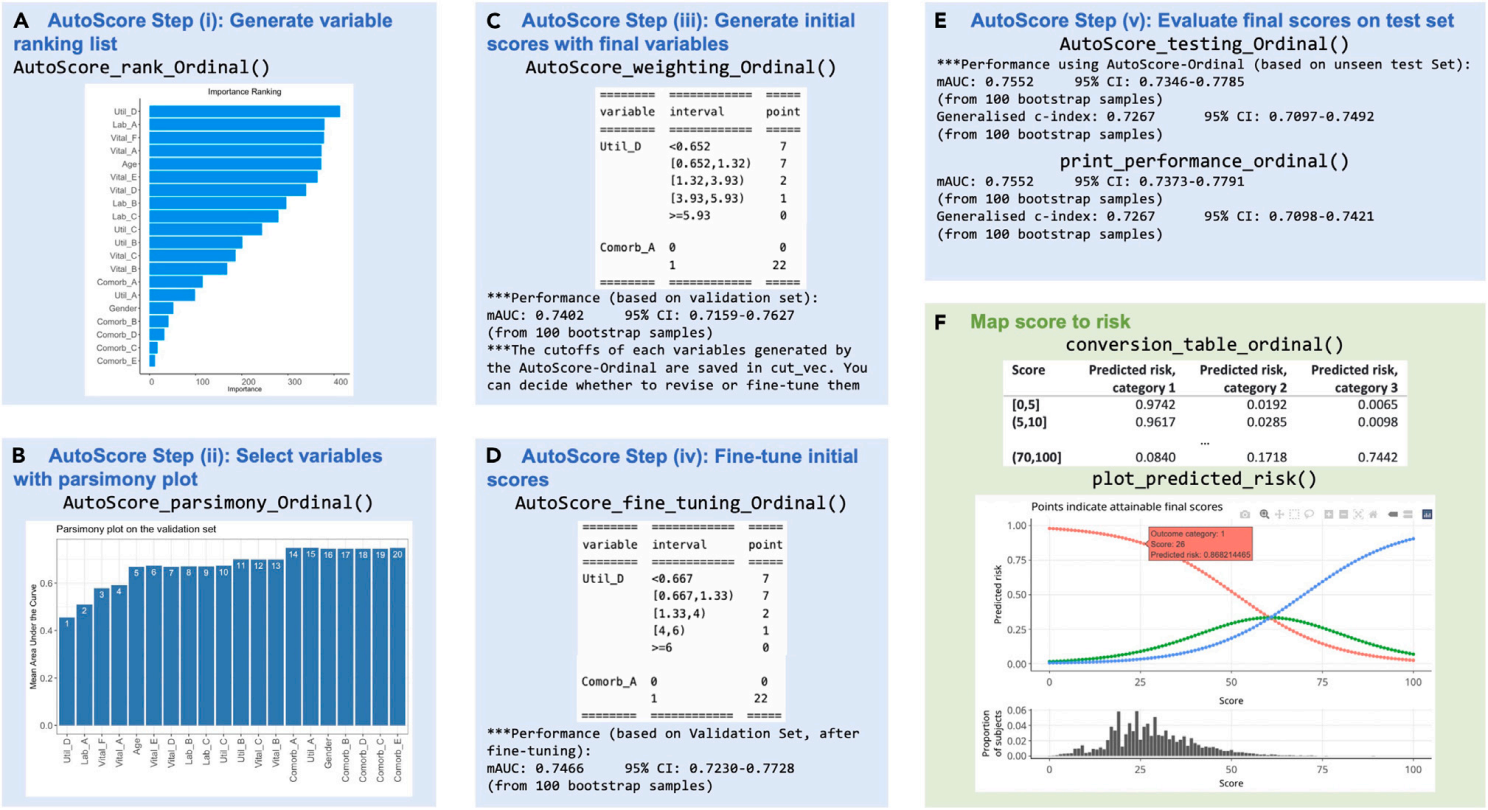
Main AutoScore functions for ordinal outcomes and corresponding output for score development and evaluation These sample datasets are simulated to demonstrate the workflow, and any results and scoring systems described here are created solely for the demonstration. (A) Variable importance from step 8, (B) parsimony plot step 9, (C) initial scoring table and performance measures from step 11, (D) fine-tuned scoring table and performance measures from step 13, (E) performance measure of the final scoring model from step 14, and (F) conversion table and visualization of predicted probabilities from steps 15 and 16.

> ranking <- AutoScore_rank(train_set = train_set, method = "rf")

***Note:*** Refer to Table 1 for a detailed description of all arguments available to each AutoScore function. The resulting variable ranking is shown in Figure 1A. Troubleshooting 5 elaborates on suggested solutions for debugging when facing some unexpected errors.

***Note:*** For survival data, please use “AutoScore_rank_Survival()” instead (see Figure 2A), which ranks variables using the random survival forest.

***Note:*** For ordinal data, please use “AutoScore_rank_Ordinal()” instead (see Figure 3A), which ranks variables using the random forest for multiclass classification.

### AutoScore step (ii): Select the best model with a parsimony plot

**Timing: < 10 min**

The second step of the AutoScore workflow helps users select a parsimonious list of variables for the final scoring model using a parsimony plot. Variable selection is flexible and can incorporate clinical knowledge and user preference in addition to model performance.

9. To generate the parsimony plot based on the variable ranking (“ranking”) from step 8, simply run the following:
  > AUC <- AutoScore_parsimony(
   train_set = train_set, validation_set = validation_set,
   rank = ranking, max_score = 100, n_min = 1, n_max = 20,
   categorize = "quantile", quantiles = c(0, 0.05, 0.2, 0.8, 0.95, 1),
   auc_lim_min = 0.5, auc_lim_max = "adaptive"
  )
  a. Key input arguments are the training and validation datasets (“train_set” and “validation_set”) and variable ranking (“ranking”). Other arguments can be adjusted to users’ needs.
  b. Refer to Table 1 for a detailed description of all input arguments.
    Performance with an increasing number of variables will be printed out on the screen, and the parsimony plot (i.e., model performance against complexity) will be available (see Figure 1B). Troubleshooting 5 elaborates on suggested solutions for debugging when facing some unexpected errors.
    ***Optional:*** Users could use the AUC for further analysis or export it as the CSV to other software for plotting.
    > write.csv(data.frame(AUC), file = "AUC.csv")
    ***Note:*** For survival data, please use “AutoScore_parsimony_Survival()” instead (see Figure 2B). To obtain a single overall performance metric in the parsimony plot, we use the integrated AUC (iAUC), a weighted average of AUC(t) over the follow-up period (the range of “label_time”).
    ***Note:*** For ordinal data, please use “AutoScore_parsimony_Ordinal()” instead (see Figure 3B, where performance is measured using mean AUC (mAUC) across dichotomized comparisons. Users have the additional option to choose the link function in the ordinal regression using the parameter “link”, which affects predictive performance. The default is link=“logit” corresponding to the commonly used proportional odds model, and users may consider “cloglog” or “probit”. The same “link” parameter must be used throughout all AutoScore functions.

10. Determine the optimal number of variables (“num_var”) based on the parsimony plot obtained in step 9. The final list of variables can be the first “num_var” (e.g., the first 6) variables:

> num_var <- 6

> final_variables <- names(ranking[1:num_var])

***Optional:*** Users can adjust the finally included variables “final_variables” based on their clinical preferences and knowledge, e.g., select the top 6 variables and the 9th and 10th variables:

> num_var <- 6

> final_variables <- names(ranking[c(1:num_var, 9, 10)])

### AutoScore step (iii): Generate initial scores with the final list of variables

**Timing: < 10 min**

This is the third step of the AutoScore workflow, which generates initial scores with the final list of variables selected in step 10.

11. Generate initial cutoff values (“cut_vec”) for all continuous variables in the list of variables from step 10 (“final_variables”), which can be fine-tuned in step 12:

> cut_vec <- AutoScore_weighting(

 train_set = train_set, validation_set = validation_set,

 final_variables = final_variables, max_score = 100,

 categorize = "quantile",

 quantiles = c(0, 0.05, 0.2, 0.8, 0.95, 1)

)

**Figure 4:**
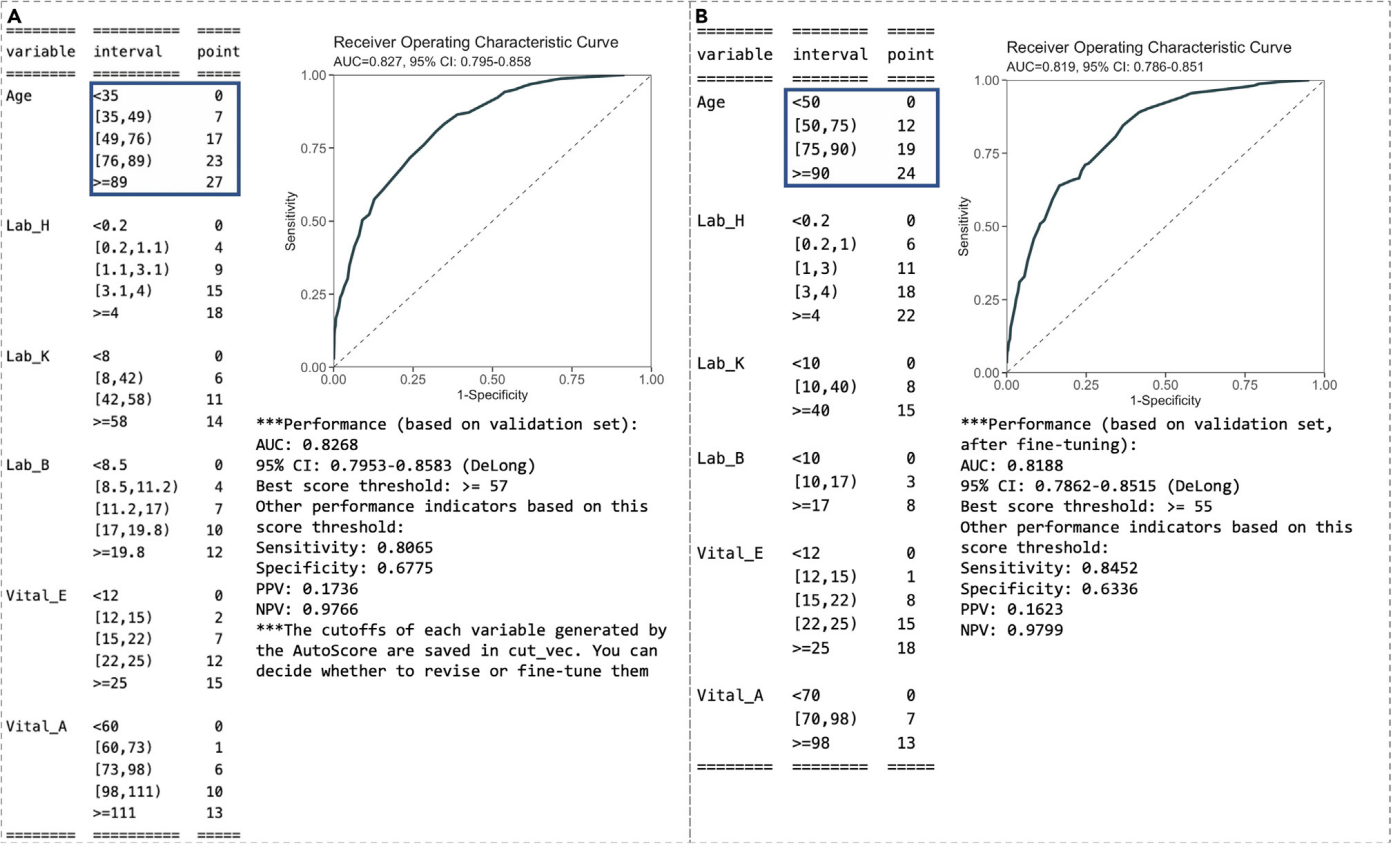
AutoScore output for intermediate scoring table evaluation and fine-tuning (A) Initial scoring table and performance measures from step 11 and (B) fine-tuned scoring table and performance measures from step 13.

The initial scoring table corresponding to the cutoff values above and the resulting intermediate performance evaluation (based on ROC evaluation for binary outcomes) will be displayed (see Figure 4A). Users can proceed to the next steps if the intermediate evaluation results are satisfactory. Otherwise, they may repeat steps 10–11 to adjust the final variable list and assess performance measures with the updated scoring table until satisfactory performance is reached. Troubleshooting 5 elaborates on suggested solutions for debugging when facing some unexpected errors.

***Note:*** For survival data, please use “AutoScore_weighting_Survival()” instead (see Figure 2C). This function requires an additional argument, “time_point”, to specify the time points at which time-dependent AUC (t) is to be evaluated.

***Note:*** For ordinal data, please use “AutoScore_weighting_Ordinal()” instead (see Figure 3C). Users have the additional option to choose the link function for the ordinal regression (see Note of step 10 for detail). Performance is measured using mAUC.

### AutoScore step (iv): Fine-tune the initial score

**Timing: < 10 min**

This step gives users an opportunity to revise the data-driven cutoff values for each continuous variable from step 11, by combining categories, rounding cutoff values up to meaningful values, or changing cutoffs according to clinical knowledge, user preference or implementation requirement.

12. After checking the initial scores and their cutoff values, users may revise the cutoff values for each continuous variable using the codes as follow.

> cut_vec$Age <- c(50, 75, 90)

> cut_vec$Lab_H <- c(0.2, 1, 3, 4)

> cut_vec$Lab_K <- c(10, 40)

> cut_vec$Lab_B <- c(10, 17)

> cut_vec$Vital_A <- c(70, 98)

***Note:*** This step is optional.

13. Run the following command to regenerate the scoring table with the updated “cut_vec” from step 12 (or the original data-driven “cut_vec” from step 11 if step 12 is skipped).

> scoring_table <- AutoScore_fine_tuning(

 train_set = train_set, validation_set = validation_set,

 final_variables = final_variables, cut_vec = cut_vec,

 max_score = 100

)

The updated scoring systems and performance based on the validation set are reported (see Figure 4B). For example, the cutoff values for age are updated from default quantile-based values to 50, 75 and 90, as specified in step 12 (indicated by blue rectangles in Figure 4), and the points for age categories are updated by retraining the model.

If the intermediate evaluation results for the current scoring system (i.e., cutoff values, score values, variables, etc.) are satisfactory, users may proceed with testing in the next step. Otherwise, users may repeat steps 12–13 to revise fine-tuning or steps 10–13 to refine not only cutoff values but also the variable list, until satisfactory performance is achieved.

***Note:*** For survival data, please use “AutoScore_fine_tuning_Survival()” instead (see Figure 2D), with an additional “time_point” argument for time points to evaluate the time-dependent AUC(t) at.

***Note:*** For ordinal, please use “AutoScore_fine_tuning_Ordinal()” instead (see Figure 3D), with an additional “link” argument to specify the link function for ordinal regression. Performance is evaluated using mAUC with 95% bootstrap CI (computed from “n_boot=100” bootstrap samples by default).

### AutoScore step (v): Evaluate final risk scores on the test dataset

**Timing: < 10 min**

This step is to evaluate the final scoring system based on the unseen testing dataset.

**Figure 5:**
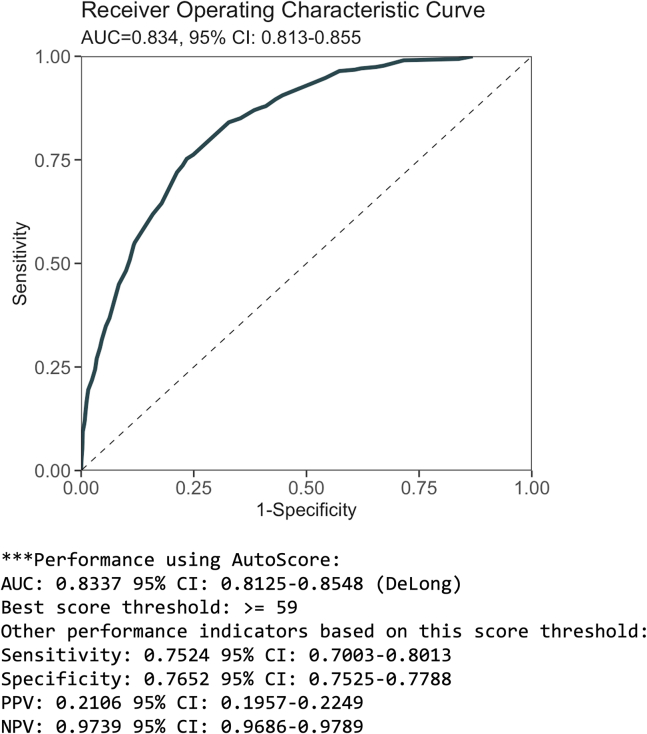
Performance measure of the final scoring model on the test set from step 14

14. Using the scoring table (“scoring_table”) generated from step 13, run the following command to generate predicted scores (“pred_score”) for each subject in the testing set (“test_set”) and print out the performance indicators (and/or performance curves, including ROC curve). The testing performance is shown in Figure 5.

> pred_score <- AutoScore_testing(

 test_set = test_set, final_variables = final_variables,

 cut_vec = cut_vec, scoring_table = scoring_table,

 threshold = "best", with_label = TRUE

)

***Optional:*** Use “print_roc_performance()” to generate the performance under different score thresholds (e.g., 90).

> print_roc_performance(pred_score$Label, pred_score$pred_score, threshold = 90)

***Note:*** For survival data, please use “AutoScore_testing_Survival()” instead (see Figure 2E), with an additional “time_point” argument for time points to evaluate the time-dependent AUC(t) at.

***Note:*** For ordinal, please use “AutoScore_testing_Ordinal()” instead (see Figure 3E), with an additional “link” argument to specify the link function for ordinal regression. In addition to mAUC, a generalized c-index is reported for the test set with 95% CI computed from “n_boot=100” bootstrap samples by default. Users can also apply “print_performance_ordinal()” to predictions to print mAUC with or without the generalized c-index (see Figure 3E).

### Map score to risk

**Timing: < 10 min**

This step describes how to map risk scores to predicted probabilities and visualize the probabilities.

15. Map risk scores to predicted probabilities using the following conversion table.

**Figure 6:**
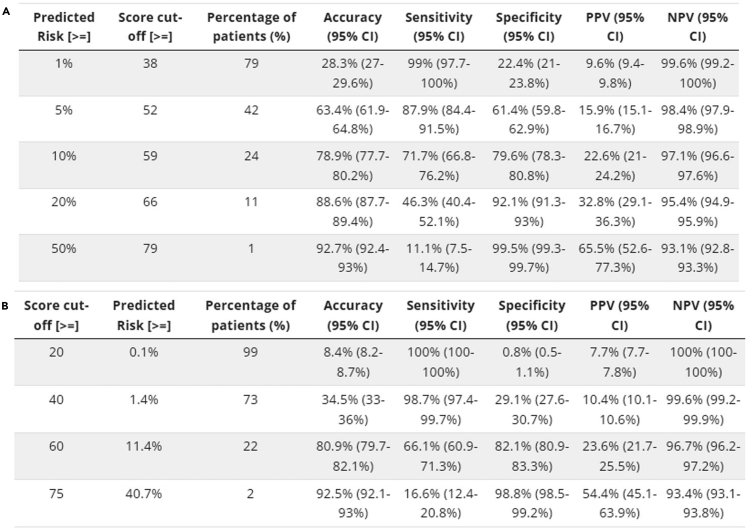
Conversion tables for binary outcomes Conversion tables generated by cut-offs in (A) predicted risks or (B) score values based on the test data.

***Note:*** For binary outcomes, users can generate conversion tables (with predictive performance measures) for specific levels of risk (e.g., 0.01, 0.05, 0.1, 0.2, 0.5) or score thresholds (e.g., 20, 40, 60, 75) using the commands below. Corresponding outputs are shown in Figures 6A and 6B, respectively. The tables are printed as text output, and users can copy and paste the tables as Excel tables when using appropriate column delimiters.

> conversion_table(pred_score, by ="risk",

     values = c(0.01,0.05,0.1,0.2,0.5))

> conversion_table(pred_score, by = "score", values = c(20,40,60,75))

***Note:*** For survival data, please use “conversion_table_survival()” instead, which reports predicted survival probabilities and selected time points (“time_point”) using specified score thresholds (“score_cut”) (see Figure 2F).

***Note:*** For ordinal data, please use “conversion_table_ordinal()” instead, which reports predicted probabilities of being in each ordinal category using specified score thresholds (“score_breaks”) (see Figure 3F).

**Figure 7:**
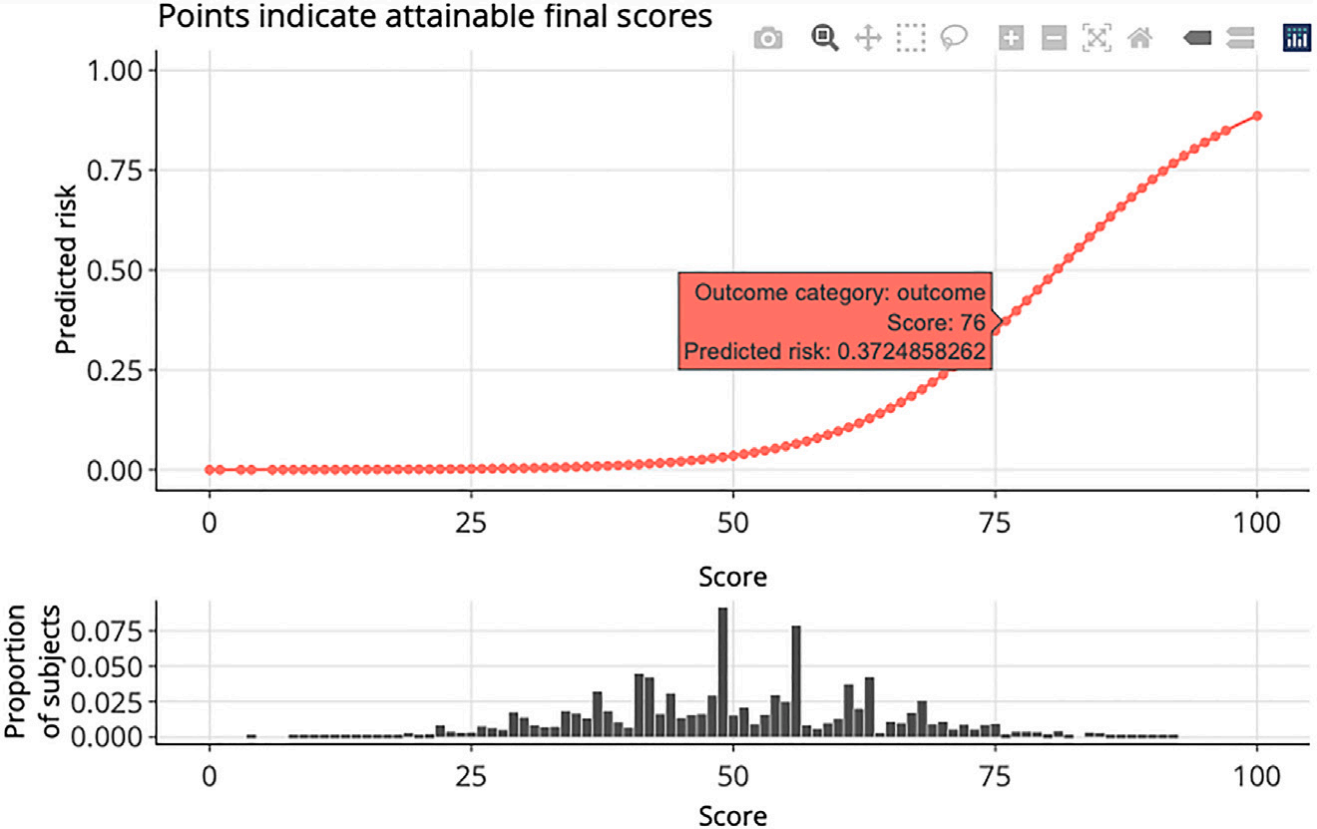
Predicted risk corresponding to risk scores for a binary outcome

16. The predicted risk corresponding to risk scores can be visualized using an interactive figure (see Figure 7 for screenshot). Users can use the built-in toolbar to zoom in for closer inspection or download it as a PNG file.

> plot_predicted_risk(pred_score = pred_score, max_score = 100,

      final_variables = final_variables,

      scoring_table = scoring_table)

***Note:*** For survival data, the Kaplan-Meier curve can be plotted using the “plot_survival_km()” function with selected score thresholds (“score_cut”). See Figure 2F.

***Note:*** For ordinal data, the same function (“plot_predicted_risk()”) can be used to visualize predicted risk for each category in an ordinal outcome. See Figure 3F.

## Expected outcomes

AutoScore can seamlessly generate risk scores using a parsimonious set of variables for different types of clinical outcomes, which can be easily implemented and validated in clinical practice. Moreover, it enables users to build transparent and interpretable clinical scores quickly in a straightforward manner. It has been extensively used in different clinical applications, e.g., for general risk assessments in the emergency department,^22^^,^^23^^,^^35^^,^^36^ and for prediction of disease-specific outcomes in specific patient cohorts.^24^^,^^25^^,^^26^^,^^37^^,^^38^^,^^39^^,^^40^^,^^41^

## Limitations

This protocol has some limitations. First, we did not provide detailed instructions for data preprocessing, as it often requires domain knowledge specific to the clinical question. Users are highly recommended to consult domain experts on the processing of raw data, outcome definition, and outlier detection and removal before importing data into AutoScore. Additionally, the final scoring system should be evaluated based on domain knowledge to ensure meaningful interpretation. Further studies are required to prepare a scoring system for clinical deployment and evaluate its feasibility for clinical implementation. Furthermore, although this protocol has covered binary, survival and ordinal outcomes, which are common in clinical studies, continuous outcomes are not included. If a continuous outcome can be meaningfully categorized into a few categories, users may analyze it as an ordinal outcome using the current AutoScore package following the steps in this protocol. Future work will investigate the feasibility of extending the scoring system to handle continuous clinical outcomes.

## Troubleshooting

### Problem 1

Fail to install the AutoScore package due to errors when installing dependencies in step 1.

### Potential solution

Ensure R version 3.5.0 or later is installed. Users are recommended to use the latest stable version of R available. When an installation error is reported for a dependent package of AutoScore, note down the name of that package, restart the R session and manually install the package using the following command:

> install.packages("<package_name>")

where <package_name> is to be replaced by the actual name of the dependency package. When the installation completes, restart the installation of AutoScore using the command in step 1.

### Problem 2

Fail to go through due to high memory usage when working with large clinical datasets, especially in steps 8 and 9.

### Potential solution

When working with large datasets, the R session may lag or abort when the maximum memory is exceeded, although this is not likely when working with typical clinical datasets. For users’ reference, when working with the “sample_data” in this protocol that has 20,000 observations and 21 variables, the memory usage was generally between 400Mb to 1Gb.

Users can easily monitor memory usage by using RStudio, which shows current memory usage and the size of large objects in the Environment panel for convenient management. Users can remove large objects to free up memory if they are no longer needed in the current session, for example, the “sample_data” and “out_split” objects after splitting data in step 7 by using the following R command:

> rm(sample_data, out_split)

Variable ranking using the random forest (i.e., step 8) can be memory- and time-consuming when working with large training sets, and an error message “Error: vector memory exhausted (limit reached?)” will be displayed if there is insufficient memory for this task. In such cases, users can consider using fewer trees (i.e., a smaller value for “ntree”) for this step, or to use a smaller training set that can sufficiently represent the full dataset.

### Problem 3

Fail to go through AutoScore data checks in step 3, i.e., “check_data()” for binary outcomes, “check_data_survival()” for survival outcomes, or “check_data_ordinal()” for ordinal outcomes.

### Potential solution

The warning or error messages explain why the dataset is not ready to be analyzed using AutoScore, and users need to address them as instructed, which we describe in detail below. Users should rerun the “check_data()” function (or “check_data_survival()” or “check_data_ordinal()”, as appropriate) after resolving each error or warning message until all data problems are resolved.

Error message “for this dataset: There is no dependent variable 'label' to indicate the outcome.” from “check_data()” indicates that the binary outcome variable is absent from the current dataset or is present but not correctly named. Users must either add the outcome to the dataset with the name ‘label’, or rename the outcome to ‘label’. Similar error messages from “check_data_survival()” and “check_data_ordinal()” indicate the absence of survival outcomes (“label_time” for time and “label_status” for status) and ordinal outcomes (“label”), respectively.

The warning message “Please keep outcome label variable binary” from “check_data()” or “check_data_ordinal()” indicates that users need to convert the outcome “label” to “factor” data type. The warning message “Please keep outcome status variable binary” from “check_data_survival()” indicates that users need to convert the status variable “label_status” to “factor” data type.

The following warning messages regarding independent variables are common to all three data checking functions.

The warning message “Special character detected in variable names” indicates that variable names in the current dataset (which will be listed after the warning message) contains special characters. Users should change the mentioned variable names, e.g., by replacing special characters by “_”.

If warned, “Too many categories (>10) in variables: ‘[variable name]’”, users need to reduce the number of categories for variables listed after this warning message so that they have less than 10 categories.

If the warning message “Variables coded as [variable type] instead of factor: ‘[variable name]’”, users should convert variables listed after this warning message to appropriate data types, e.g., “factor” for categorical variables and numeric for “continuous” variables.

If a warning message reports the presence of missing entries in the current dataset, users should inspect the number and proportion of missing entries reported after this warning message, and decide whether to handle the missing values manually via methods like exclusion or imputation before applying the AutoScore workflow or to keep them as “NA” (if they are informative and prevalent enough). If the user would like to preserve the missingness, they can directly move to the next step because AutoScore can automatically handle the missingness by treating them as a new category “Unknown”.

### Problem 4

R fails to consider missing entries as “NA”, especially in steps 5 and 8.

### Potential solution

If missing entries in a dataset “data.csv” are represented by characters such as a white space, “/”, “NA” or “N.A.”, by default, they will be read into R as meaningful strings and will not be considered by R as missing. Users can use the following command to check and understand all values present in variable “x” of dataset “data”:

> table(data$x, useNA="ifany")

To appropriately recognize special characters as missing information, users can specify the representation of missing when reading the data into R using command:

> read.csv("data.csv", na.strings = c(" ", "/", "NA", "N.A."))

### Problem 5

Encounter other errors when using the AutoScore package to process your data in different steps, especially in steps 8, 9, 11, 13 and 14.

### Potential solution

Although AutoScore is aimed to become a universal package that is compatible with any structured data, some unique data structures (e.g., with highly sparse data or uncommon data distribution) might cause errors during AutoScore processing even after the data pass the “check_data()” function. We highlight that the “check_data()” function focus on data formatting and missing issues. Users should carefully inspect the input data (by using “compute_descriptive_table()” or other R functions) before building models using AutoScore to avoid unreliable findings and prevent errors. We provide the following steps for users to debug and proceed:

• Make sure all “check_data()” requirements have been fulfilled and all warnings and errors have been fully resolved. Confirm this by rerunning “check_data()” after resolving each error or warning.
• Carefully read the R error messages and try to narrow them down to a specific variable that might have caused the error.
• If users manage to identify the variable causing the error, inspect this variable in greater detail (e.g., variable distribution, sparsity, outliers, etc.) to find feasible remedies (e.g., manually categorizing continuous variables, combining categories in categorical variables, excluding problematic variables from analysis, etc.).
• If this error persists, or if the error message is unclear, report the error to https://github.com/nliulab/AutoScore/issues with descriptive statistics for relevant variables (preferably with sample data, if possible), to help us better understand the error.
• After receiving the error report, our team will provide targeted suggestions for you. This will also help us improve the package and user experience for future researchers.

## Resource availability

### Lead contact

Further information and requests for resources and reagents should be directed to and will be fulfilled by the lead contact, Nan Liu (liu.nan@duke-nus.edu.sg).

### Materials availability

This study did not generate new unique reagents.

## Data Availability

For complete details on the use and execution of this protocol, please refer to https://nliulab.github.io/AutoScore/. The full code repository is available at https://github.com/nliulab/AutoScore, and the current version is archived at Zenodo: https://zenodo.org/record/7813554#.ZDQO8i8Rrx8.
